# Early detection of cognitive impairment by natural language among outpatients and community dwellers

**DOI:** 10.1093/geroni/igaa057.3291

**Published:** 2020-12-16

**Authors:** Eri Kiyoshige, Mai Kabayama, Yasushi Takeya, Yoichi Takami, Shuko Takeda, Yasuyuki Gondo, Hiromi Rakugi, Kei Kamide

**Affiliations:** 1 Osaka University Graduate School of Medicine, Suita, Osaka, Japan; 2 Osaka University, Suita, Osaka, Japan

## Abstract

Background: Although early detection of cognitive decline has a significant relation to improving the quality of life of dementia patients, this early detection has been difficult due to requires of neuropsychological tests which people generally take when they notice their cognitive impairment. The timing of patients’ notice was reported to be worse cognitive decline already, thus, we aimed to determine if cognitive impairment from a short interview by using Natural Language Processing approach. Methods: The present study used cross-sectional analysis among elderly outpatients and community-dwelling elderly from Septuagenarians, Octogenarians, Nonagenarians Investigation with Centenarians (SONIC) study. Cognitive decline was assessed by Telephone Interview of Cognitive Status for Japanese (TICS-J) and modeled as a binary outcome (cut-off <33 points). Natural language data was collected by semistructured interviews about health conditions and cognitive orientation in space, time, and place. We used an open-source text segmentation library to parse natural language text into bag-of-words and term frequency-inverse document frequency (TF-IDF) representations. Results: There were 38 (19.9%) outpatients and 153 (80.1%) community dwellers, and 60 (31.4%) participants were defined as cognitive impairment. The maximized TF-IDF score was 0.49 in cognitive orientation in time questions. In this question, participants without cognitive impairment could not calculate the score. There were no significant differences in TF-IDF scores between participants with and without cognitive impairment. Conclusions: Elderly without cognitive impairment might not have an episode about cognitive orientation in time, and this may help for early detection of cognitive impairment

